# Pathogenicity and Transmissibility of North American Triple Reassortant Swine Influenza A Viruses in Ferrets

**DOI:** 10.1371/journal.ppat.1002791

**Published:** 2012-07-19

**Authors:** Subrata Barman, Petr S. Krylov, Thomas P. Fabrizio, John Franks, Jasmine C. Turner, Patrick Seiler, David Wang, Jerold E. Rehg, Gene A. Erickson, Marie Gramer, Robert G. Webster, Richard J. Webby

**Affiliations:** 1 Division of Virology, Department of Infectious Diseases, St. Jude Children's Research Hospital, Memphis, Tennessee, United States of America; 2 Department of Pathology, St. Jude Children's Research Hospital, Memphis, Tennessee, United States of America; 3 Veterinary Diagnostic Laboratory (NCVDL) System, North Carolina Department of Agriculture, Raleigh, North Carolina, United States of America; 4 Veterinary Diagnostic Laboratory, College of Veterinary Medicine, University of Minnesota, St. Paul, Minnesota, United States of America; Johns Hopkins University - Bloomberg School of Public Health, United States of America

## Abstract

North American triple reassortant swine (TRS) influenza A viruses have caused sporadic human infections since 2005, but human-to-human transmission has not been documented. These viruses have six gene segments (PB2, PB1, PA, HA, NP, and NS) closely related to those of the 2009 H1N1 pandemic viruses. Therefore, understanding of these viruses' pathogenicity and transmissibility may help to identify determinants of virulence of the 2009 H1N1 pandemic viruses and to elucidate potential human health threats posed by the TRS viruses. Here we evaluated in a ferret model the pathogenicity and transmissibility of three groups of North American TRS viruses containing swine-like and/or human-like HA and NA gene segments. The study was designed only to detect informative and significant patterns in the transmissibility and pathogenicity of these three groups of viruses. We observed that irrespective of their HA and NA lineages, the TRS viruses were moderately pathogenic in ferrets and grew efficiently in both the upper and lower respiratory tracts. All North American TRS viruses studied were transmitted between ferrets via direct contact. However, their transmissibility by respiratory droplets was related to their HA and NA lineages: TRS viruses with human-like HA and NA were transmitted most efficiently, those with swine-like HA and NA were transmitted minimally or not transmitted, and those with swine-like HA and human-like NA (N2) showed intermediate transmissibility. We conclude that the lineages of HA and NA may play a crucial role in the respiratory droplet transmissibility of these viruses. These findings have important implications for pandemic planning and warrant confirmation.

## Author Summary

North American triple reassortant swine (TRS) influenza A viruses have caused sporadic human infections, but human-to-human transmission has not been established. We wished to elucidate potential human health threats posed by the TRS viruses and to identify determinants of virulence in the TRS and closely related 2009 H1N1 pandemic viruses. We used a ferret model to evaluate the pathogenicity and transmissibility of North American TRS viruses with the HA and NA antigenic proteins of swine viruses and of human viruses. We observed that the North American TRS viruses grew efficiently in both the upper and lower respiratory tracts and caused moderate pathogenicity in ferrets. The viruses were readily transmissible via direct contact, irrespective of their HA and NA lineages. However, transmissibility via respiratory droplets was substantially greater when the viruses carried the HA and NA of human influenza A viruses rather than of swine influenza A viruses. Because ferrets are a useful model of human influenza infection, this finding helps to predict features that increase the risk to human health.

## Introduction

For nearly 70 years, swine influenza virus in North America was relatively stable, dominated by the classical-swine H1N1 (cH1N1) subtype [1]. However, H3 seasonal human influenza A viruses were circulating at low frequency in U.S. swine [2]. In 1998, influenza epidemiology in North American swine changed dramatically with the emergence of double-reassortants (combining gene segments of cH1N1 and seasonal human H3N2 influenza A viruses) and triple-reassortants (adding gene segments from avian influenza lineages). The triple-reassortants gained predominance in North American swine and continued to evolve, further reassorting with cH1N1 and contemporary seasonal human influenza viruses [3], [4]. All of the currently circulating North American triple-reassortant swine (TRS) influenza A viruses contain a similar constellation of internal genes (avian PA and PB2, human PB1, and classical swine-lineage M, NP, and NS), but their surface glycoproteins are derived from different lineages (classical swine-lineage H1 and N1 and seasonal human-lineage H1, H3, N1 and N2).

Sporadic infections with TRS H1N1 (swine-like HA and NA) and H1N2 (swine-like HA, human-like NA) viruses have been reported in humans exposed to swine in North America [5]. Some have included severe lower respiratory tract disease and diarrhea. H3N2 (human-like HA and NA) TRS viruses have also been isolated from humans [6], [7], [8]. In 2009, TRS viruses with human-like H1 and N1 (closely related to A/Brisbane/59/2007 [H1N1]) caused cough, fever, nasal congestion, rhinorrhea, sneezing, malaise, and dizziness in humans [9]. These symptoms were very similar to those caused by the 2009 H1N1 pandemic viruses, which possessed six gene segments (PB2, PB1, PA, HA, NP, and NS) closely related to those of North American TRS viruses [10]. However, unlike the 2009 H1N1 pandemic viruses, the TRS viruses were not reported to be transmissible among humans.

Despite extensive recent studies of the pathogenicity and transmissibility of pH1N1 viruses in different animal models [11]–[14], there is very little information of this kind about North American TRS viruses. A/swine/Kansas/77778/2007 (H1N1), a triple reassortant similar to H1N1 viruses that infected humans and pigs at an Ohio county fair in 2007, was isolated from swine herds in the Midwestern United States. This virus is highly virulent in swine and is readily transmitted to sentinel pigs [15]. TRS virus A/Swine/Texas/4199-2/98 (H3N2) was also shown to be transmissible from infected swine to direct-contact swine and from them to a second group of direct-contact swine [16]. Belser and co-workers found two North American H1N1 TRS viruses (with swine-like HA and NA) isolated from humans to be pathogenic in mice [17]. In ferrets, these viruses showed pathogenicity similar to that of 2009 pandemic H1N1 influenza virus but less efficient transmissibility [18]. We have shown that the TRS virus A/swine/Arkansas/2976/02 (H1N2) and the Eurasian avian-like swine virus A/swine/Hong Kong/NS29/09 (H1N1) are not transmissible via respiratory droplets in ferrets [19]. The TRS virus A/swine/Guangdong/1222/2006 (H1N2) and the Eurasian avian-like swine virus A/swine/Fujian/204/2007 (H1N1) were recently shown not to be transmissible by direct contact in guinea pigs [20]. Very recently, Pearce et al. demonstrated that H3N2 TRS viruses isolated from humans were efficiently transmitted via respiratory droplets (RD) in ferrets [8].

Most of the North American TRS viruses belong to three subtypes: H1N1, H1N2, and H3N2; H1 and N1 are of the classical swine or seasonal human lineages, while H3 and N2 are of seasonal human lineage only. TRS viruses with human-like HA and NA have recently become the predominant influenza viruses isolated from swine in the US (St. Jude swine influenza surveillance program, unpublished data), but their transmissibility has not been tested in the ferret model. Ferrets are an established small-animal model that appears to recapitulate the pathogenicity and transmissibility of human seasonal influenza A viruses [11]–[13] and the poor human transmissibility of H5 and H7 avian influenza A viruses [21]–[23].

In the present study, we used the ferret model to evaluate the pathogenicity and transmissibility of three distinct groups of North American TRS viruses (H1N1 viruses with classical swine-like HA and NA; H1N2 viruses with classical swine-like HA but human-like NA; and H1N1, H1N2, and H3N2 viruses with human-like HA and NA) and of the Eurasian avian-like swine virus A/sw/Italy/1369-7/1994 (H1N1) (Italy/94). Because a limited number of ferrets could be used, the study was designed to detect patterns in the transmissibility and pathogenicity of TRS viruses that, once confirmed, will have important implications for pandemic preparedness. Italy/94 virus was less efficiently transmissible than the North American TRS viruses. The North American TRS viruses, regardless of their HA and NA lineages, were readily transmissible to co-housed (direct contact, DC) ferrets, while viruses with human-like HA and NA or with human-like NA alone showed enhanced transmission via respiratory droplets.

## Results

### Pathogenicity

**Figure 1:**
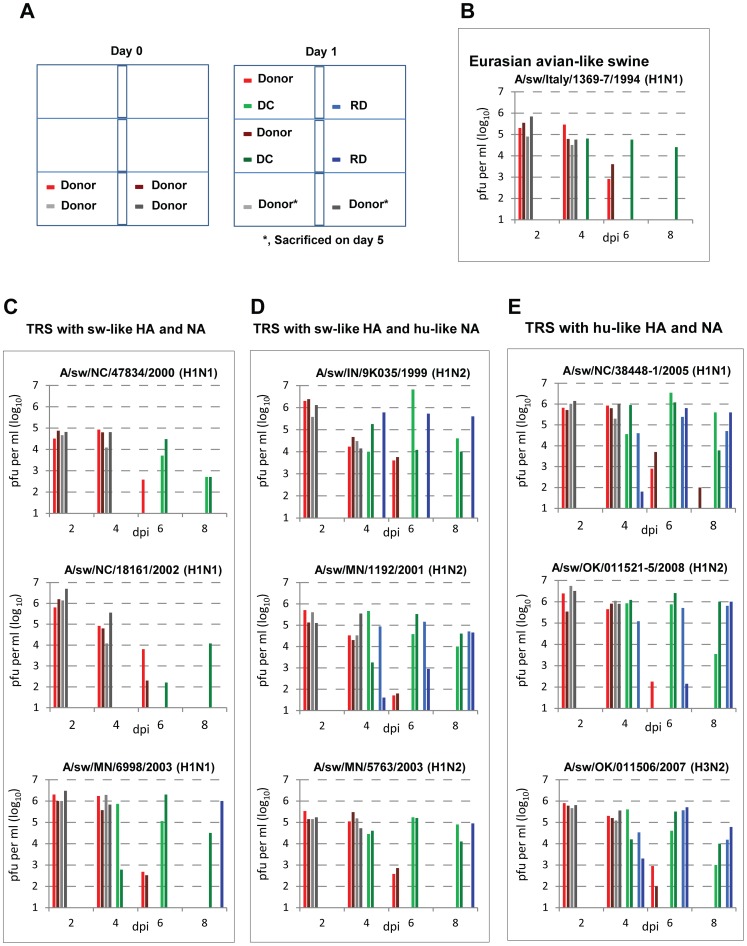
Transmissibility of North American TRS viruses in ferrets. A) Isolator scheme. Four donor ferrets were inoculated with 10^6^ pfu of virus and housed in the lower cages. The next day, two donor ferrets were moved into separate cages, each containing one naïve DC ferret. Two naïve RD ferrets were housed separately in cages adjacent to the donor ferrets but separated by grills to allow unobstructed airflow while preventing direct contact. Nasal washes were collected from inoculated (red and gray bars) and contact (green, DC; blue, RD) animals on the indicated days p.i. for virus titration. Inoculated animals remaining in the lower cages were euthanized on day 5 p.i. for tissue studies (nasal washes were collected on days 2 and 4 p.i.). B–E) Nasal wash titers of B) Eurasian avian-like swine, C) TRS viruses with sw-like HA and NA, D) TRS viruses with sw-like HA but hu-like NA, and E) TRS viruses with hu-like HA and NA. Color coding is shown in panel A. Day 1 p.i. = day 0 post-exposure. sw, swine; hu, human.

Ferrets inoculated with 10^6^ pfu of each virus (a dose previously found to result in consistent infection [11]–[13], [18], [22]) showed only mild clinical signs of illness, mild to moderate weight loss (∼3% to 9%), and infrequent sneezing (Table 1). Among the ferrets that lost weight, weight loss was maximal during days 4 to 6 pi, and then weight began to increase. Ferrets that did not lose weight showed no significant change until day 4 pi and then began gaining weight. A few inoculated ferrets (e.g., two inoculated with Italy/94) gained weight continuously, starting on day 1. A few ferrets (mainly those inoculated with TRS viruses with human-like HA and NA) had elevation of body temperature (maximum increase, 1.5°C). We observed no significant lethargy and no ruffled fur. Infectious virus was observed in nasal washes until approximately day 6 post-inoculation (p.i.), with peak titers on day 2 p.i. TRS virus A/sw/NC/47834/2000 (H1N1, with swine-like HA and NA) caused the least nasal virus shedding (mean peak titer, 5×10^4^ pfu/ml vs. 10^6^ pfu/ml for the other two viruses in this group) (Fig. 1).

**Table 1:** Clinical signs and virus replication in inoculated ferrets.

Viruses	Clinical signs		Virus titer (log_10_ pfu/ml)^b^			
	Weight loss No/total (mean a max. % loss)	Sneezing (No/total)	Nasal turbinates	Trachea upper	Trachea lower	Lung^c^
**Eurasian avian like swine:**						
A/sw/Italy/3169-7/1994 (H1N1)	1/4 (5)	0/4	–, –	–, 5.3	–, 4.5	3.3, 4.6
**TRS with sw-like HA and NA**						
A/sw/NC/47834/2000 (H1N1)	2/4 (5)	0/4	4.6, 4.7	3.7, 2.8	3.2, 2.3	3.5, 2.9
A/sw/NC/18161/2002 (H1N1)	2/4 (9)	1/4	4.6, –	3.5, –	2.8, 1.7	3.5, 2.6
A/sw/MN/6998/03 (H1N1)	2/4 (6)	1/4	2.9, 3.9	4.8, 4.5	4.9, 5.0	6.0, 2.6
**TRS with sw-like HA and hu-like NA**						
A/sw/IN/9K035/99 (H1N2)	3/4 (5)	2/4	4.7, 5.1	2.6, 5.3	3.8, 4.2	5.3, 5.3
A/sw/MN/1192/01 (H1N2)	2/4 (9)	2/4	4.5, 1.3	5.6, 4.1	4.7, 5.5	1.8, 6.0
A/sw/MN/5763/03 (H1N2)	1/4 (3)	1/4	5.0, 4.7	4.5, 2.6	3.8, 2.8	3.5, 2.3
**TRS with hu-like HA and NA**						
A/sw/NC/38448-1/2005 (H1N1)	1/4 (5)	1/4	5.7, 2.9	4.8, 3.2	4.3, 3.8	5.2, 4.7
A/sw/OK/011521-5/2008 (H1N2)	2/4 (5)	2/4	5.3, –	5.7, 6.3	6.0, 5.8	5.1, 5.8
A/sw/OK/011506/2007 (H3N2)	1/4 (7)	0/4	5.1, 4.8	4.2, 2.9	4.5, 2.8	5.1, 3.7

Sw, swine; hu, human.

–, Below the limit of detection (1.3 log_10_ pfu/ml).

a Mean (when multiple ferrets lost weight) percent maximum weight loss (ferrets not showing weight loss were excluded from this calculation).

b Values derived from two ferrets.

c Homogenates combining portions of all six lobes.

One of the TRS isolates (A/sw/IN/9K035/1999 [H1N2]) with swine-like HA but human-like NA caused peak nasal virus shedding (mean peak titer, 10^6^ pfu/ml) substantially higher than that caused by the other two viruses in this group (mean peak titers, 2×10^5^ pfu/ml and 1×10^5^ pfu/ml). TRS isolates containing human-like HA and NA caused the highest peak nasal wash titers (mean, 10^6^ pfu/ml). Infectious virus particles were identified at various titers in the lungs of all ferrets inoculated with the studied TRS viruses (Table 1). Ferrets inoculated with the Italy/94 virus had low peak nasal wash titers (mean, 1.7×10^5^ pfu/ml) and exhibited almost no clinical signs. However, infectious virus particles were obtained from the lungs of both inoculated ferrets.

These findings show that overall, North American TRS viruses grow efficiently in both the upper and lower respiratory tracts of ferrets irrespective of their HA and NA lineages and cause moderate pathogenicity, similar to the reported pathogenicity of the 2009 pandemic H1N1 viruses [11]–[14].

### Lung histopathology

**Figure 2:**
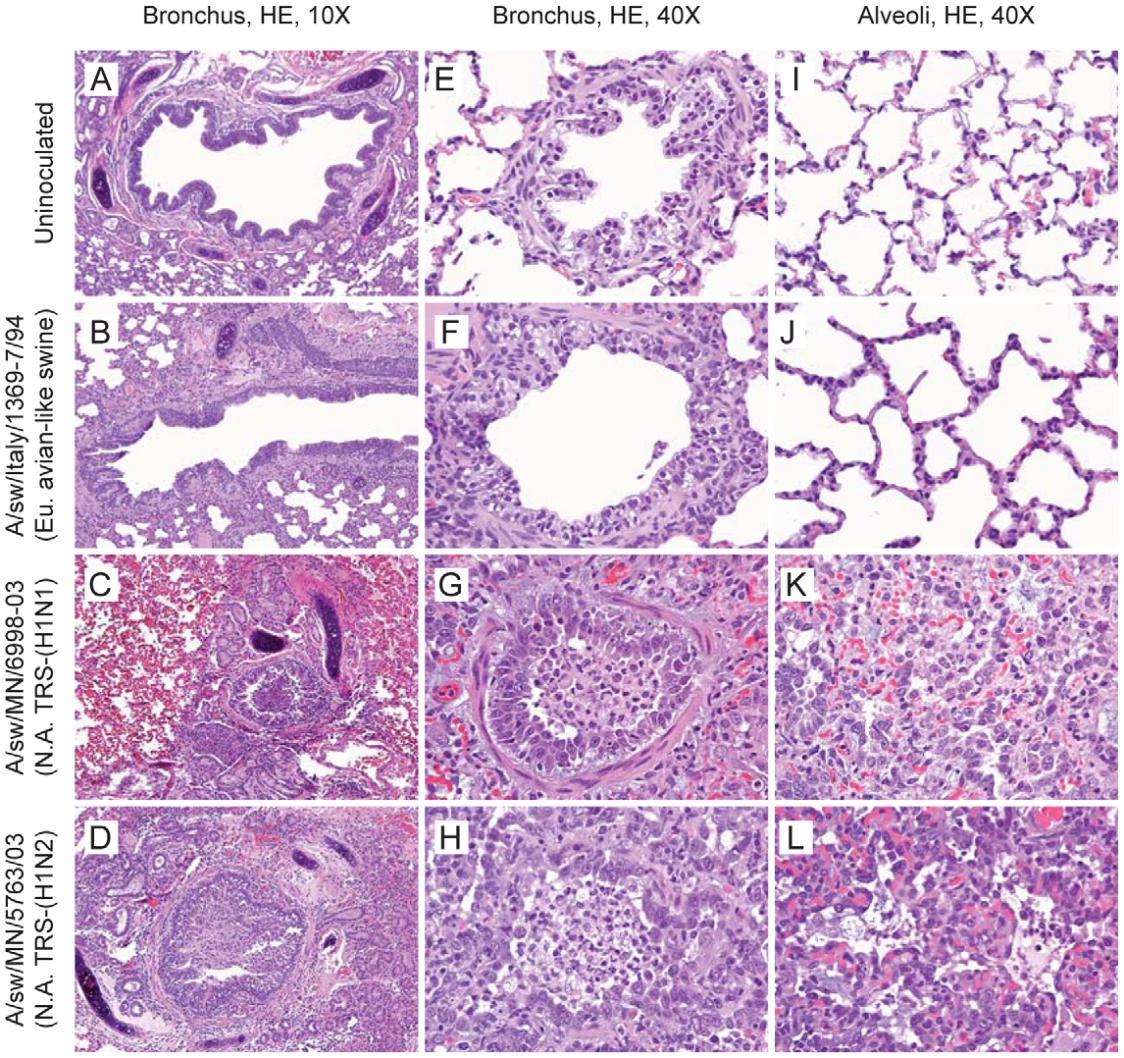
Histopathology of ferret lung tissue. Lung tissue of control (un-inoculated) and virus-inoculated ferrets was collected on day 5 p.i. Formalin-fixed, paraffin-embedded 5-µm sections were stained with hematoxylin and eosin and microscopically examined in a blinded fashion. Representative images show bronchi (A–D), bronchioles (E–H), and alveoli (I–L) from un-inoculated (A,E,I) and virus-inoculated ferrets. The two North American TRS viruses (C,G,K and D,H,L) caused bronchitis, bronchiolitis, alveolitis, and alveolar wall interstitial changes. The bronchitis featured intraluminal granulocytes and/or mucus, bronchial epithelial hyperplasia with submucosal mucus gland loss, and mixed inflammatory-cell infiltrates. The bronchiolitis featured intraluminal cellular debris, sloughed epithelial cells, and inflammatory cells (macrophages and/or granulocytes). The peribronchiolar alveoli contained mixed inflammatory cell infiltrates and foci of pneumocyte hyperplasia. The Eurasian avian-like swine virus (B,F,J) caused morphologic changes similar to those caused by the TRS viruses but far less severe.

All of the North American TRS viruses caused bronchitis, bronchiolitis, alveolitis and alveolar wall interstitial changes, with varying degrees of involvement and severity. The degree of involvement and the severity also varied to some extent in different ferrets inoculated with the same virus and in different lobes of the same lung. Fig. 2 shows representative changes. The bronchitis featured intraluminal granulocytes and/or mucus, bronchial epithelial hyperplasia with submucosal mucus gland loss, and mixed inflammatory-cell infiltrates. The bronchiolitis featured intraluminal cellular debris, sloughed epithelial cells, and inflammatory cells (macrophages and/or granulocytes) with or without bronchiolar epithelial cell necrosis and/or regenerative epithelial cell hyperplasia and hypertrophy. In the alveolitis, the alveoli surrounding the bronchioles contained a mixture of inflammatory cell infiltrates (granulocytes, lymphocytes, plasma cells and macrophages) and foci of pneumocyte hyperplasia. The interstitia (alveolar walls) were either normal or thickened by increased cellularity. Italy/94 virus caused similar morphologic changes but they were far less severe than those caused by the North American TRS viruses.

### Transmission

Unlike the 2009 H1N1 pandemic viruses, North American TRS viruses are not known to be transmissible among humans [5], [9]. To investigate factors that affect transmissibility, we assessed the transmission of different TRS viruses by direct contact (DC; in co-housed ferrets) and by respiratory droplets (RD). Italy/94 (Eurasian avian-like swine) virus was transmitted to only one of two DC ferrets and to neither RD ferret (Table 2, Fig. 1B), indicating poor transmission efficiency.

**Table 2:** Transmission of North American TRS viruses in ferrets via direct contact (co-housing) and respiratory droplets.

Viruses	Transmission (No. positive/total)			
	Direct contact		Respiratory droplets	
	Virus detection^a^	Seroconversion	Virus detection^a^	Seroconversion
**Eurasian avian like swine:**				
A/sw/Italy/3169-7/1994 (H1N1)	1/2	1/2	0/2	0/2
**TRS with sw-like HA and NA**				
A/sw/NC/47834/2000 (H1N1)	2/2	2/2	0/2	0/2
A/sw/NC/18161/2002 (H1N1)	1/2	1/2	0/2	0/2
A/sw/MN/6998/03 (H1N1)	2/2	2/2	1/2^b^	1/2
**TRS with sw-like HA and hu-like NA**				
A/sw/IN/9K035/99 (H1N2)	2/2	2/2	1/2	1/2
A/sw/MN/1192/01 (H1N2)	2/2	2/2	2/2	2/2
A/sw/MN/5763/03 (H1N2)	2/2	2/2	1/2^b^	1/2
**TRS with hu-like HA and NA**				
A/sw/NC/38448-1/2005 (H1N1)	2/2	2/2	2/2	2/2
A/sw/OK/011521-5/2008 (H1N2)	2/2	2/2	2/2	2/2
A/sw/OK/011506/2007 (H3N2)	2/2	2/2	2/2	2/2

Sw, swine; hu, human.

a In nasal wash specimens.

b Delayed transmission: virus detected on day 7 post-exposure (day 8 p.i.).

Among the TRS viruses with swine-like HA and NA, A/sw/NC/47438/2000 (H1N1), which had the lowest mean peak nasal wash titer (≤10^5^ pfu/ml), was transmitted only by direct contact (2/2 DC, 0/2 RD) (Fig. 1C). Although the A/sw/NC/18161/2002 virus replicated efficiently (mean peak nasal wash titer, 10^6^ pfu/ml) in donor ferrets, transmission was observed in only one of two DC ferrets and in neither RD ferret. Infectious A/sw/MN/6998/2003 was detected on day 7 post-exposure (p.e.) (day 8 p.i.) in the nasal wash of one of the two RD ferrets. Overall, only one of the three viruses in this group was transmitted via RD, to one of two ferrets; the remaining two viruses were not transmitted via RD. Therefore, TRS viruses with swine-like HA and NA were poorly transmitted via RD. Poor RD transmission of TRS viruses containing swine-like HA and NA has been reported previously [16].

The TRS viruses with human-like HA and NA replicated efficiently (mean peak nasal wash titer, ∼10^6^ pfu/ml) and were efficiently transmitted (2/2 DC, 2/2 RD) in ferrets (Fig. 1E and Table 2), consistent with a recent report on the RD transmissibility of TRS viruses containing human-like HA and NA (H3N2) [8]. The transmission efficiency of TRS viruses with swine-like HA and human-like NA (N2) varied from strain to strain. Among these viruses, A/sw/MN/1182/2001 was as efficiently transmitted (2/2 DC, 2/2 RD) as TRS viruses with human-like HA and NA. In contrast, infectious A/sw/MN/5763/2003 and A/sw/IN/9K035/1999 viruses were detected in the nasal wash of only one of two RD ferrets (Fig. 1D, Table 2). A/sw/MN/5763/2003 virus was first detected in a RD ferret on day 7 p.e., while the other two viruses in this group were first detected on day 3 p.e.

Taken together, these results suggest that in the ferret model, 1) transmissibility of Italy/94 (Eurasian avian-like swine) virus is poor, 2) North American TRS viruses are readily transmissible by direct contact irrespective of their HA and NA lineages, and 3) transmissibility via RD of TRS viruses with swine-like HA and NA, swine-like HA but human-like NA, and human-like HA and NA is poor, moderate, and efficient, respectively.

### Growth kinetics in MDCK cells

**Figure 3:**
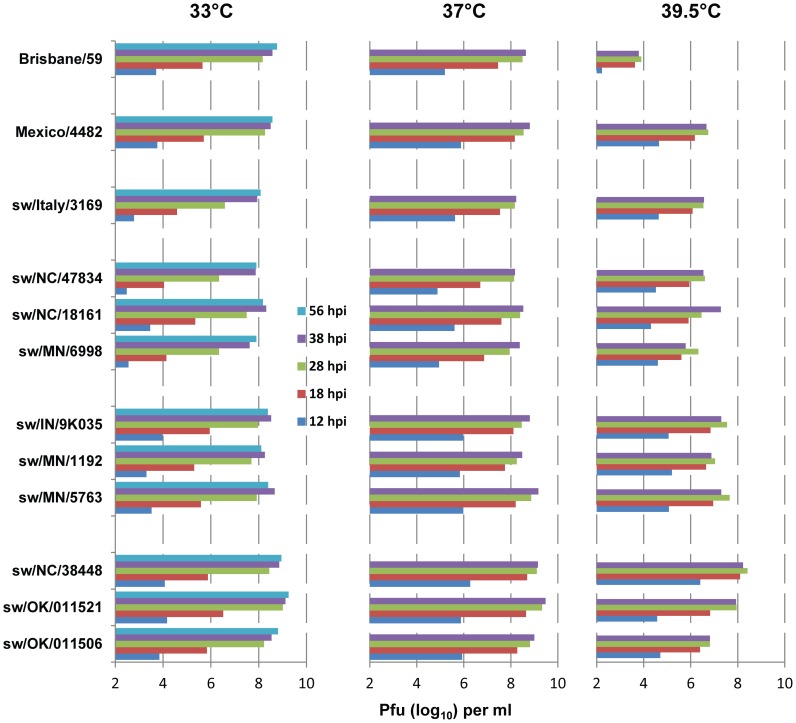
Growth characteristics of North American TRS viruses in MDCK cells. Cells were inoculated with the respective viruses at an MOI of 0.001 and incubated at the indicated temperatures. At the indicated h p.i., supernatants were harvested and virus was titrated by pfu assay. At the higher temperatures (37°C and 39.5°C), cytopathic effects caused detachment of most infected cells from the dish after 38 h p.i.; therefore, virus release was not examined beyond that point. Values are the mean of two independent experiments performed in duplicate (n = 4).

Our results show that unlike seasonal H1N1 viruses, whose replication is reported to occur primarily in the upper respiratory tract [11]–[13], TRS viruses grow efficiently in ferret lungs and cause substantial lung pathology, similar to that reported for pandemic H1N1 viruses [11]–[13]. As the temperature is higher in the lower than the upper respiratory tract, ability to grow at a higher temperature might favor virus growth in the ferret lung. To better understand the pathogenicity of the swine isolates studied, we examined the multi-cycle growth kinetics of these swine viruses, of seasonal human H1N1 virus A/Brisbane/59/2007, and of the 2009 pandemic H1N1 virus A/Mexico/4482/2009 in MDCK cells at different temperatures. MDCK cell monolayers were inoculated with the viruses at a multiplicity of infection (MOI) of 0.001 and incubated at 33°C, 37°C, and 39.5°C. At different h p.i., supernatants were harvested and virus was titrated by pfu assay (Fig. 3). At 33°C, Italy/94 (Eurasian avian-like swine) virus and TRS viruses with swine-like HA and NA had similar growth kinetics, with peak titers of ∼10^8^ pfu/ml. TRS viruses with human-like HA and NA grew to substantially higher titers (∼10^9^ pfu/ml), while TRS viruses with swine-like HA and human-like NA grew to titers similar to those of seasonal H1N1 or 2009 pH1N1 viruses (10^8^–10^9^ pfu/ml, Fig. 3). At 37°C, although final yield increased only slightly, replication of all viruses was accelerated, as noted by significantly higher titers at 12 and 18 h p.i. At 39.5°C, replication of all swine viruses and of the 2009 pH1N1 virus A/Mexico/4482/2009 was less (by a factor of 10 to 100) than their replication at 37°C. However, the reduction of virus titer at 39.5°C was greatest for seasonal human H1N1 virus A/Brisbane/59/2007 (6×10^3^ pfu/ml vs. 10^6^–10^8^ pfu/ml for swine and pH1N1 viruses).

At all three temperatures, TRS viruses with human-like HA and NA grew to the highest titers, whereas those with swine-like HA and NA grew to the lowest titers. These replication characteristics somewhat paralleled the viruses' overall respiratory droplet transmission efficiency.

## Discussion

Despite sporadic human infections with North American TRS influenza A viruses, their human-to-human transmission has not been established, and pathogenicity and transmission studies in animal models have been very limited. This study of the pathogenicity and transmissibility of North American TRS viruses containing both swine- and human-like HA and NA found that the viruses grow efficiently in both the upper and lower respiratory tracts and cause moderate pathogenicity similar to that reported for the 2009 pandemic H1N1 viruses [11]–[14]. The TRS viruses were readily transmissible by direct contact in ferrets, irrespective of their HA and NA lineages. However, RD transmissibility varied significantly with the lineages of HA and NA: TRS viruses with swine-like HA and NA, swine-like HA but human-like NA, and human-like HA and NA were transmitted poorly, moderately, and efficiently, respectively, via respiratory droplets.

Multiple viral factors, including HA receptor specificity, human-specific amino acid residues (e.g., 627K/701N) in PB2, and balance between HA and NA, are known to influence transmission and pathogenicity in humans [24]–[28]. Like the 2009 pH1N1 viruses, all TRS viruses studied here have avian-origin PB2 containing 627E and 701D. However, SR polymorphism (590S and 591R) within pH1N1 PB2 was shown to partly compensate for the absence of 627K in polymerase activity and virus replication in human A549 cells, suggesting that this polymorphism plays a role in efficient growth of pH1N1 viruses in the human upper respiratory tract [29]. E627K substitution in PB2 was later shown not to alter the growth of pH1N1 virus in MDCK cells at 33°C, 37°C, or 39°C or to significantly alter its virulence and replication in mouse and ferret lung tissues [30]–[32]. Importantly, the PB2 of Italy/94 (Eurasian avian-like swine) virus, containing 627E/701D, was associated with less lung pathology and transmissibility; it also lacks the SR polymorphism, instead containing 590G/591Q, which were shown to reduce the polymerase activity of 2009 pH1N1 virus by 50% [29]. Like pH1N1, all of our TRS viruses contain the avian-specific amino acids 627E and 701D and the SR polymorphism (590S and 591R) in PB2, (with the exception of A/sw/NC/47834/2000, which contains 590S and 591Q and showed lower nasal wash titers and poor transmissibility in ferrets), indicating involvement of other factors in their differential RD transmission. In our experiments, although the TRS viruses with human-like HA and NA replicated efficiently (mean peak nasal wash titer, ∼10^6^ pfu/ml) and were efficiently transmitted in ferrets (Fig. 1E and Table 2), transmission did not always parallel virus shedding. For example, the TRS virus A/sw/NC/18161/2002 (H1N1), with swine-like HA and NA, had an average peak nasal wash virus titer (10^6^ pfu/ml) similar to those of viruses with human HA and/or NA but was least transmissible (1/2 DC, 0/2 RD) in ferrets. In contrast, the TRS virus A/sw/MN/1192/2001(H1N2), with swine-like HA and human-like NA, had a significantly lower average peak nasal wash virus titer (2×10^5^ pfu/ml) than A/sw/NC/18161/2002 (10^6^ pfu/ml) but was transmitted efficiently in ferrets.

H1 HA of North American swine isolates comprises four distinct phylogenetic groups, H1α (cH1N1), H1β (TRS H1N1-like), H1γ (TRS H1N2-like), and H1δ (human-like) [33], [34]. The HAs of some recent TRS H1N1 isolates with swine-like HA and NA are closely related to H1γ (Fig. S1), as are pH1N1 HAs. Recently, two TRS viruses isolated from humans, A/Texas/14/08 (H1N1, H1β) and A/Ohio/2/07 (H1N1, H1γ), showed poor aerosol transmissibility in ferrets [18]. In our study, RD transmission of H1N1 TRS viruses with swine-like H1β HA and swine-like NA was poor in ferrets. However, the H1N2 TRS viruses A/sw/MN/1182/2001 (H1N2) and A/sw/IN/9K035/1999 (H1N2) which possess H1γ HA, were readily transmitted via respiratory droplets, suggesting that human-like NA (N2) is responsible for RD transmissibility of North American TRS viruses containing swine-like H1γ HA. However, studies using reassortant RG viruses are needed to confirm that human-like NA (N2) can enhance the RD transmissibility of TRS viruses containing swine-like HA. Our group and, more recently, others have demonstrated that pH1N1 NA and M can enhance RD transmission of TRS viruses in ferrets [19], [35]. However, others have shown that in a guinea pig model that Eurasian avian-like swine virus (A/sw/Fujian/204/2007[H1N1]) NA and M are not sufficient to alter the non-transmissibility of North American TRS virus (A/sw/Guandong/1222/2006[H1N2]) and that the HA and NS of 2009 pandemic H1N1 virus (A/Beijing/7/2009) contributes to its transmissibility [20].

We found that the North American TRS viruses grew well at 39.5°C. The TRS viruses yielded 2×10^6^ to 2×10^8^ pfu/ml at 39.5°C, while seasonal human H1N1 A/Brisbane/59/07 yielded only 6×10^3^ pfu/ml under identical growth conditions (Fig. 3). This finding may explain the growth of TRS viruses in ferret lungs. At 33°C and 37°C, the yield of seasonal human virus (2×10^8^ pfu/ml) and TRS viruses was similar. Interestingly, at all three temperatures the growth kinetics of the TRS viruses with swine-like HA was very similar to that of the 2009 pandemic A/Mexico/4482/2009 (H1N1) virus (Fig. 3). It was reported that unlike seasonal H1N1 viruses, whose replication is primarily restricted to the upper respiratory tract, the 2009 pandemic H1N1 viruses replicated efficiently in ferret lungs [11]–[13]. A recent study found that replacing the HA of seasonal H1N1 virus A/New York/312/2001 with the HA of 2009 pH1N1 A/Mexico/4108/2009 (swine-like) virus reduced surfactant protein D binding and increased lung pathology in mice, although it did not increase lung virus titers [36]. In our experiments, TRS viruses with either swine-like or human-like HA caused significant lung pathology, yielded high lung virus titers, and replicated efficiently in MDCK cells at 39.5°C. As the lower respiratory tract is warmer, ability to grow at a higher temperature may be responsible at least in part for the efficient lung growth and significant lung pathology in ferrets. Further studies of growth characteristics in primary human respiratory epithelial cells, which may more closely recapitulate the human respiratory tract, are warranted. The molecular determinants of the efficient *in vitro* growth of the TRS and pH1N1 viruses at 39.5°C and the relation of this growth to their efficient replication in ferret lungs are of interest for future studies.

TRS viruses containing human-like HA and NA showed the highest RD transmissibility in the ferret model, likely reflecting a higher rate of replication (high virus titers *in vitro* and *in vivo*), efficient release of progeny virions in the presence of human-like NA, and efficient re-infection in the presence of human-like HA. In contrast, this group of TRS viruses causes only sporadic human infection, indicating a possible limitation of the ferret model. Importantly, however, ferrets used in this study (and in all transmissibility studies) were farm-raised and sero-negative for influenza A viruses. Acquired immunity within the human population, in addition to viral and environmental factors, plays a critical role in human-to-human transmission of influenza A viruses [37]. It is possible that vaccination and pre-exposure to human-like HA and NA in the human population inhibits the spread of this group of viruses in humans. Unlike HA-specific antibodies, NA-specific antibodies do not prevent influenza virus infection, and NA immunity is referred to as infection-permissive [38]. However, humoral immunity induced by NA can markedly reduce virus replication and release, moderating the severity and duration of illness [39]–[42]. Human infections with H1N2 TRS viruses containing swine-like HA have been reported [5], but in humans, unlike the ferret experimental model, transmission is likely to be partially inhibited by NA-mediated immunity to seasonal influenza viruses, including H3N2. Therefore, the rapid worldwide human spread of pH1N1 may be partially explained by its acquisition of Eurasian avian-like swine virus NA and M in a North American TRS genetic background (with swine-like HA) in two ways. First, its RD transmissibility could have been enhanced by the presence of the Eurasian NA and M and second, its pandemic potential could have been enhanced by the absence of immunity to the swine-like HA and NA in the human population.

The pandemic 2009 H1N1 virus is now the predominant human H1N1 influenza virus worldwide. Vaccine against seasonal human H1N1 does not offer significant protection against 2009 pH1N1, and therefore seasonal H1N1 has been replaced by 2009 pH1N1 (with North American swine-like HA and Eurasian swine-like NA) in the World Health Organization's recommended trivalent vaccine. TRS viruses are reported to cause severe lower respiratory tract disease and diarrhea in humans [5]. Here we have shown that unlike seasonal H1N1 (e.g., A/Brisbane/59/2007), whose replication is reported to be restricted primarily to the upper respiratory tract [11], TRS viruses grow efficiently in the lung and cause substantial lung pathology in ferrets. Most importantly, we have shown that H1N1 TRS viruses with human-like HA and NA (which reportedly do not cross-react with antibody to 2009 pandemic H1N1 [43]) are efficiently transmitted in the ferret model, indicating that in the absence of pre-exposure or vaccination to seasonal H1N1, these viruses may be transmissible among humans, especially young children, and therefore are a public health concern.

## Materials and Methods

### Animals

Four- to six-month-old male ferrets (Triple F farms, Sayre, PA; Marshall Farms, Hazle Township, PA) that were serologically negative for currently circulating influenza viruses by hemagglutination inhibition (HI) assay were used. All animal experiments were conducted in an Animal Biosafety Level 2+ (level 2 with enhanced biocontainment for pandemic H1N1 influenza A virus) facility at St. Jude Children's Research Hospital, in compliance with the policies of the National Institutes of Health and the Animal Welfare Act and with the approval of the St. Jude Children's Research Hospital Animal Care and Use Committee.

### Cells and viruses

MDCK cells were maintained in Dulbecco modified Eagle's medium (DMEM; Invitrogen Corporation, Grand Island, NY) supplemented with 10% fetal bovine serum and antibiotics-antimycotic (Sigma, St. Louis, MO; 100 U/ml penicillin, 100 µg streptomycin, and 0.25 µg amphomycin per ml). Stock viruses were propagated in embryonated chicken eggs at 37°C for 48 h. All isolates underwent a limited number of passages in eggs to maintain their original properties. The genome sequences of A/sw/OK/011521-5/2008 (H1N2), A/sw/OK/011506/2007 (H3N2), A/sw/IN/9K035/99 (H1N2), and A/Mexico/4482/2009 (H1N1) have been described previously [43]–[45]. Our group previously performed genome sequencing and GenBank submission of A/Brisbane/59/2007 (H1N1). For all other viruses, complete genomes were sequenced as described previously [46] and sequences were submitted to GenBank. Sequence alignment and phylogenetic analysis of HA and NA (Fig. S1) and the other six gene segments (data not shown) confirmed Italy/94 (H1N1) as a Eurasian avian-like swine virus and the other nine swine viruses as North American triple reassortants of the subtypes and HA and NA lineages shown in Table 1.

### In vitro virus growth kinetics and plaque assay

MDCK cell monolayers were inoculated with viruses at an MOI of 0.001 and maintained in virus growth medium (modified Eagle's medium [Invitrogen] containing 1% BME vitamins [Sigma], 0.2% BSA [Calbiochem], 1.6 mg/ml NaHCO_3_ [Invitrogen], antibiotics-antimycotic [Sigma], and 1.0 µg/ml of tosylsulfonyl-phenylalanyl-chloromethyl-ketone [TPCK]-treated trypsin [Sigma]) (1.6 ml per well in 6-well plates) at different temperatures. Plaque assays were done in MDCK cells in the presence of 1.0 µg/ml TPCK-treated trypsin in agarose overlay medium (virus growth medium containing 0.0015% DEAE-dextran hydrochloride [prepared from dextran of mean molecular weight 500,000; Sigma] and 0.9% ultra-pure low-melting-point agarose [Invitrogen]), as reported previously [47]. After incubation at 37°C for 60 h the plaques were visualized by staining with 0.1% crystal violet solution containing 10% formaldehyde.

### Pathogenicity and transmission experiments

Baseline body weight and temperature were documented before inoculation or contact exposure. Four donor ferrets per virus were housed in the lower cages of isolators (configured as shown in Fig. 1A) in ABSL2+ facilities. Air was uniformly circulated at 52 to 57 air changes per hour. Ambient temperature and relative humidity were maintained. The donor ferrets were lightly anesthetized with isoflurane and inoculated with 10^6^ pfu of virus in 0.5 ml PBS (250 µl per nostril). The next day (day 1 p.i.), two donor ferrets were moved into separate cages, each containing one naïve direct contact (co-housed) ferret (Fig. 1A). For respiratory droplet transmission assay, two naïve ferrets were housed separately in cages adjacent to the donor ferrets but separated by double-layered (3 inches apart) grills to allow unobstructed airflow but prevent direct contact. A borazine gun (Zero Toys, Concord, MA) was used to ensure smooth air flow from the left cages to the right cages within each isolator. The donor and recipient ferrets remained housed together from day 1 p.i to day 20 p.i. Weight, temperature, and clinical signs (sneezing, lethargy, and ruffled fur) were recorded every other day for 14 days. Nasal washes were collected on days 2 (donors only), 4, 6, and 8 p.i. by flushing nostrils with total 1.0 ml PBS, and pfu titers were determined in MDCK cells. The two donor animals remaining in the lower cages (Fig. 1A) were euthanized on day 5 p.i. for histopathology of the lung and virus titration of the nasal turbinates, trachea (upper and lower), and lung.

### Histopathology

Lung tissue was collected from control (un-inoculated) and virus-inoculated ferrets on day 5 p.i., fixed in 10% neutral buffered formalin, and embedded in paraffin. 5-µm sections were stained with hematoxylin and eosin and examined by microscopy in a blinded fashion. Histopathology was examined separately for the bronchi, bronchioles, alveoli and alveolar interstitial walls of each lung lobe.

### Serologic tests

Serum samples were collected from ferrets at day 20 p.i., treated for 18 h at 37°C with receptor-destroying enzyme, heat-inactivated at 56°C for 30 min, and tested by HI assay with 0.5% packed chicken red blood cells as described previously [48].

### GenBank virus sequence accession numbers

CY058484-91, CY098465-72, CY098473-80, CY098481-88, CY098489-96, CY098497-504, CY098505, CY098506-12, and CY098513-20 for A/Brisbane/59/2007 (H1N1), A/swine/MN/1192/2001 [H1N2], A/swine/NC/47834/2000 [H1N1], A/swine/MN/6998/2003 [H1N1], A/swine/MN/5763/2003 [H1N2], A/sw/Italy/1369-7/1994 [H1N1], A/Mexico/4482/2009 (H1N1), A/swine/NC/38448-1/2005 [H1N1], and A/swine/NC/18161/2002 [H1N1], respectively.

## Supporting Information

Figure S1 Phylogenetic tree of the A) HA and B) NA gene segments based on nucleotide sequences from GenBank. Evolutionary history was inferred by using the neighbor-joining method. The percentage of replicate trees in which the associated taxa clustered in the bootstrap test (500 replicates) are shown next to the branches. Only bootstrap values >60 are shown. The tree is drawn to scale, with branch lengths indicating the evolutionary distances used to infer the phylogenetic tree. The evolutionary distances (the number of base substitutions per site) were computed by using the Kimura 2-parameter method. All ambiguous positions were removed for each sequence pair (pairwise deletion option). Evolutionary analyses were conducted by using the MEGA5 program. sw, swine; Eu sw, Eurasian avian-like swine; hu, human; pan, pandemic. •, Viruses used in this study.
(EPS)
